# Broadband Transmission EPR Spectroscopy

**DOI:** 10.1371/journal.pone.0059874

**Published:** 2013-03-21

**Authors:** Wilfred R. Hagen

**Affiliations:** Department of Biotechnology, Delft University of Technology, Delft, The Netherlands; Instituto de Tecnologica Química e Biológica, UNL, Portugal

## Abstract

EPR spectroscopy employs a resonator operating at a single microwave frequency and phase-sensitive detection using modulation of the magnetic field. The X-band spectrometer is the general standard with a frequency in the 9–10 GHz range. Most (bio)molecular EPR spectra are determined by a combination of the frequency-dependent electronic Zeeman interaction and a number of frequency-independent interactions, notably, electron spin – nuclear spin interactions and electron spin – electron spin interactions, and unambiguous analysis requires data collection at different frequencies. Extant and long-standing practice is to use a different spectrometer for each frequency. We explore the alternative of replacing the narrow-band source plus single-mode resonator with a continuously tunable microwave source plus a non-resonant coaxial transmission cell in an unmodulated external field. Our source is an arbitrary wave digital signal generator producing an amplitude-modulated sinusoidal microwave in combination with a broadband amplifier for 0.8–2.7 GHz. Theory is developed for coaxial transmission with EPR detection as a function of cell dimensions and materials. We explore examples of a doublet system, a high-spin system, and an integer-spin system. Long, straigth, helical, and helico-toroidal cells are developed and tested with dilute aqueous solutions of spin label hydroxy-tempo. A detection limit of circa 5 µM HO-tempo in water at 800 MHz is obtained for the present setup, and possibilities for future improvement are discussed.

## Introduction

The phenomenon of electron paramagnetic resonance (EPR), or electron spin resonance (ESR), is at the basis of a well established spectroscopy widely used in multiple disciplines including, e.g., physics, chemistry, biology, for the characterization of electronic structure of open-shell systems with electron spin S ≠ 0 [1]–[3]. The word ‘resonance’ in EPR refers to the quantum-mechanical phenomenon of transition between molecular spin energy levels induced by microwave radiation (typically: gigahertz frequency or centimeter wavelength). The actual spectroscopic experiment also involves a classical, macroscopic resonance phenomenon because the sample is held inside a cavity which is constructed as a single-frequency, fundamental-mode resonator cell whose quality factor insures high radiation energy density at the sample to overcome the intrinsically low concentration sensitivity associated with the detection of molecular energy differences of the order of the thermal energy kT.

With very few exeptions the resonator has always been a key component of the EPR experiment ever since its inception nearly seven decades ago [4]. Also the modern EPR spectrometer is a single-frequency instrument, and its ability to produce spectra depends on the possibility of tuning molecular energy level differences by means of an external static magnetic field of variable strenght. However, microwave-frequency variation is an important and often essential strategy in EPR analysis for the disentanglement of multiple molecular magnetic interactions some of which are independent of the external magnetic field B, while others are linearly dependent on B. This has led to the cumbersome and costly practice of multi-frequency EPR as the serial deployment of a small number of physically separate spectrometers each one with its own operating frequency. Replacement by a single, frequency-tunable spectrometer would not only be very convenient, but it would also allow for the collection of two-dimensional frequency-field data at a much higher frequency-axis density.

Molecular EPR spectroscopy without a resonator has been occasionally explored previously. In the early ‘80 s Bramley and Strach reported on a zero-field (i.e. without a magnet) reflection spectrometer working with a sweep oscillator based on a backward wave tube with a typical tuning range of 1–8 GHz, and employing a non-resonant coaxial sample cell (of unspecified dimensions) with the sample in lieu of the dielectric, which gave good room-temperature frequency-swept spectra of 1.5 mol% Mn(II) in magnesiumammoniumsulfate powder [5]. This corresponds to a concentration of circa 50 mM high-spin paramagnet, and so for sensitivity reasons in a later version of the zero-field spectrometer the non-resonant coaxial setup was replaced with a loop-gap resonator [6]. A broadband, field-scanning, non-resonant reflection spectrometer was described in 1989 by Rubinson, who used a plate-ended brass coaxial tube and a 5.4–12.5 GHz signal generator [7]. The instrument afforded only a very weak signal at 9 GHz from 770 mM Mn(II) in pure hydrated manganese chloride. In later work the cell was modified into a quarter-wavelength truncated-line probe (a resonator) for work at <1 GHz [8], although a non-resonant version has also been used for FT-EPR at 0.2–0.4 GHz [9]. All in all it appears that the historical verdict on non-resonant sample cells for cw (continuous wave)-EPR has been to drop the subject from further consideration in view of the poor signal-to-noise values attained. Transmission cells have been used in high-frequency (≥ ≈100 GHz) high-field EPR with low concentration sensitivity; these are oversized waveguide structures which sustain a multiplicity of excited modes [10].

The present work describes the development of theory, basic design principles, and applications for field-swept transmission EPR spectroscopy based on an instrument in which the single-frequency reflection resonator has been replaced by a continuously frequency-tunable, broad-band, fundamental mode transmission cell. A central design goal of the present work is to combine the added versatility of microwave source tunability over a broad frequency range with a concentration sensitivity comparable to that of extant conventional single-frequency spectrometers.

## Results

### Theory of Transmission EPR Spectroscopy

The magnetic susceptibility tensor of a compound is a complex quantity
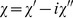
(1)when measured in response to a field with time-varying components. In magnetic resonance spectroscopy the field B is the vector sum of a static field B_0_ from the external magnet and a perpendicular time-varying field B’ from the RF source. At the resonance frequency ν = c/λ = ω/2π the dynamic susceptibility is detected as an average power dissipated in the spins



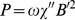
(2)(e.g., [11]). Conventional EPR spectrometers with field modulation produce spectra as graphs of δχ″/δB (arbitrary units) versus B (gauss or tesla).

The magnetic susceptibility is related to the complex magnetic permeability μ = μ′-iμ″ and the relative magnetic permeability µ_R_ as
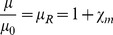
(3)with complex components



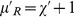
(4)




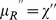
(5)in which
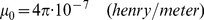
(6)is the permeability of free space.

In field theory [12] the response of a compound to a transverse electromagnetic (TEM) wave is defined by a plane wave propagation constant γ and a characteristic impedance Z_0_. The propagation constant
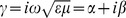
(7)in which ε = ε′−iε″ = ε_R_ε_0_ is the complex electric permittivity of the medium,
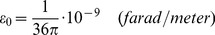
(8)is the permittivity of free space, α is the attenuation factor, and β is the phase factor of the wave. The characteristic impedance, or the ratio of the electric field E and magnetic field H, is



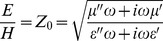
(9)The equivalent definition of characteristic impedance Z_0_ in distributed transmission-line theory [12] is in terms of a ratio of voltage across a line segment over current through the segment
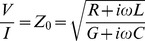
(10)with series resistance R, series inductance L, shunt capacitance C, and shunt conductance G. The propagation constant becomes



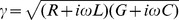
(11)The equivalence implies that the EPR effect in transmission spectroscopy (μ″≠0 for the paramagnet) will be detected as a resistive loss serial to the resistance of the line conductors; it also announces a key experimental problem of non-resonant power dissipation by conductance of lossy diamagnetic hosts (ε″>>0 for the diamagnetic host and/or the paramagnet) in particular in aqueous solutions.

For a particular transfer-line geometry the propagation constant and the characteristic impedance provide the design parameters in terms of mode sustainability, attenuation, and power handling capacity. We will work this out to obtain expressions for EPR absorption in a coaxial or quasi-coaxial cell in which part of the dielectric insulator consists of a paramagnet or diluted paramagnet.

**Figure 1:**
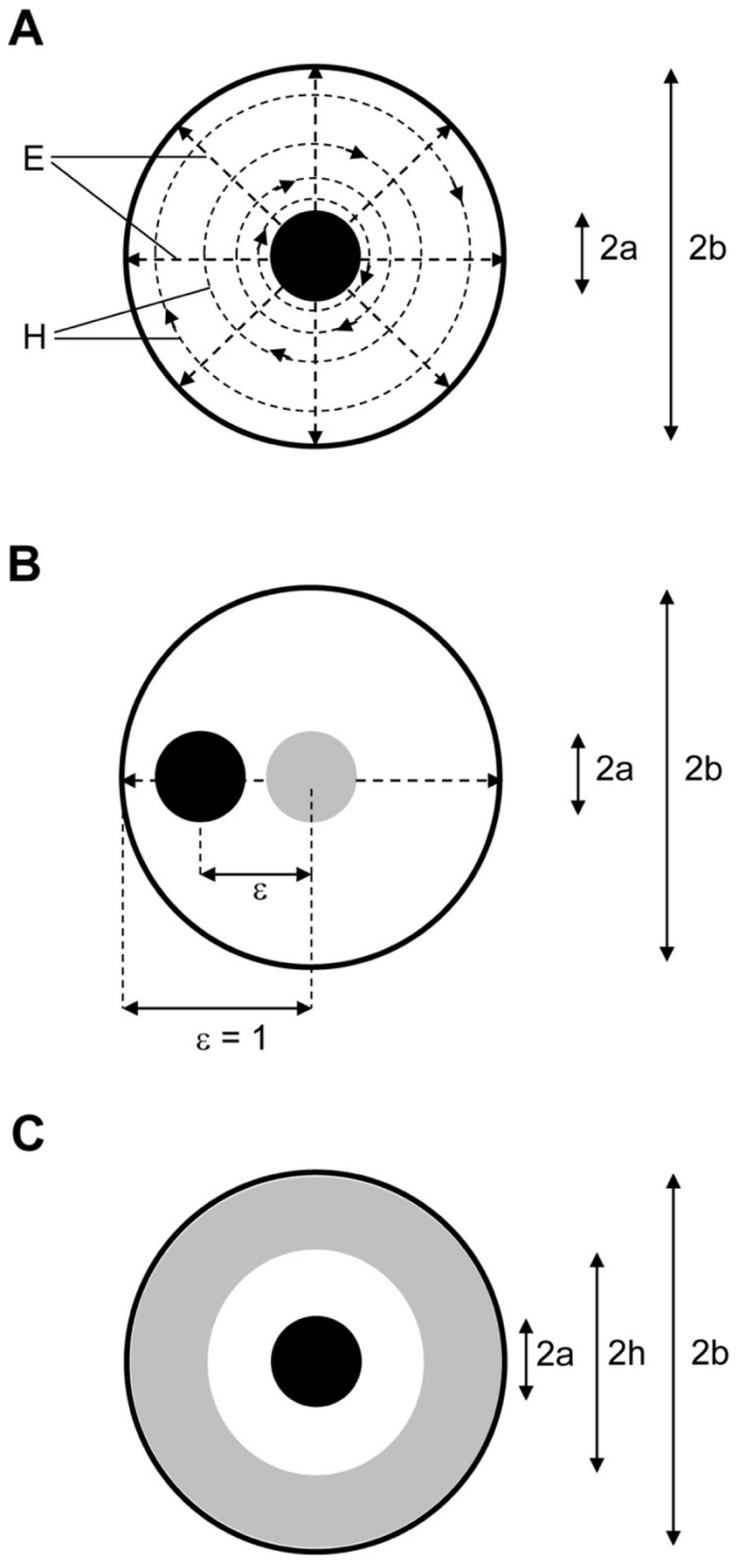
Cross sections of coaxial line. (A) Outline of electric (E) and magnetic (H) field lines; note radial the decrease in H-density. The outer diameter of the innner conductor is 2a and the inner diameter of the outer conductor is 2b. (B) Definition of eccentricity of an off-axis inner conductor. (C) Illustration of a coaxial cell in which a paramagnetic sample (white area) is contained in a diamagnetic holder (grey area).



The characteristic impedance for a loss-less coaxial line is (e.g., [13])
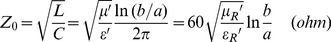
(12)in which 2b is the inner diameter of the outer conductor and 2a is the diameter of the inner conductor (Fig. 1a). With the universal standard of Z_0_ = 50 ohm for RF equipment we can set up a spectrometer in which the transmission cell is designed as a coaxial device under test (DUT) connected to a vector network analyzer (VNA, see below). Increased design flexibility comes from the extension that the two-conductor geometry may vary from circular coaxial to circular eccentric [14] affording
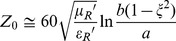
(13)with an eccentricity 0 ≤ ξ <1 (cf Fig. 1b), and from the notion that ‘the’ dielectric of the line in practice will be a (diamagnetically diluted) paramagnetic sample, s, contained in a diamagnetic cylindrical holder, h, of finite wall dimension and with an electric permittivity different from that of the sample (Fig. 1c) whose individual impedances are taken to be additive according to their fractional volume V as

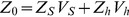
(14)The attenuation of a TEM wave in a dielectric is [12], [15]

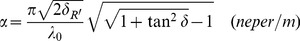
(15)in which λ_0_ is the wavelength of the microwave in vacuo,



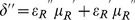
(16)




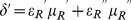
(17)and the loss tangent, tanδ, is



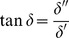
(18)With the Taylor expansion for tan^2^δ <<1
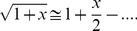
(19)and, converting from nepers to decibels by multiplication with 20log(e), the attenuation becomes



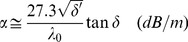
(20)This formal description encompasses a variety of physical processes of relevance for our spectroscopy including dielectric losses through the dilutant and other insulators, propagation of higher-order modes, resistive losses in the inner and outer conductors, reflective losses by impedance mismatches, and resonance attenuation via permeability absorption by the paramagnetic solute.

For this latter *extra* attenuation induced at electron paramagnetic resonance, assuming the absence of paraelectic resonance in the solute (extra resonance ε_R_″ = 0), eqn (20) becomes
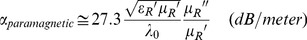
(21)


The average power from a sinusoidal field that flows through a cylindrical segment of unit length of a coaxial cable along the direction of TEM propagation is a constant (e.g., [16], p. 72):
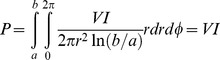
(22)whence it follows that the EPR absorption for a paramagnet homogeneously distributed over the insulator volume of a coaxial transmission cell is linear in the length, l, of the cell. It is also linear in the concentration, c, of the paramagnet since µ_R_″ was defined in terms of the volume magnetic susceptibility. Remarkably, the EPR absorption is independent of the diameter of the cell for a given characteristic line impedance (i.e. for a fixed ratio of inner and outer conductor radii b/a defining Z_0_ = 50 Ω). In an analogy with optical spectroscopy one could call this the transmission-EPR equivalent of Beer’s law, which can be written in terms of a microwave-intensity ratio as
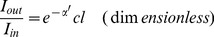
(23)or as an absorbance
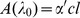
(24)in which α′ is the molar attenuation obtained by multiplying α with the number of paramagnetic particles per volume. For maximal EPR signal intensity this expression alone suggests us to make the paramagnet as concentrated as possible and to make the cell as long as geometric constraints (e.g., by the EPR magnet) would allow. Its independence of cell diameter also implies that one can maximally economize on amount of sample by minimizing the diameter of the cell down to the technical limits of miniaturization. The inverse wavelength relation in Eq 21 implies that S = 1/2 EPR signal intensity is proportional to the microwave frequency.

This simple picture becomes a complex design problem of trade-offs when combined with the other, non-resonance related, phenomena of attenuation. Of paramount importance are the losses in the dielectric(s), which, under the assumptions that µ_R_′ ≈ 1 and µ_R_″ ≈ 0, are usually given as (e.g., [16])
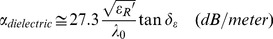
(25)with tanδ_ε_ = ε_R_″/ε_R_′. Since this α is also linear in the length of the cell (and independent of its diameter) we can only increase the length to a point where overall output power becomes too weak to be handled reliably by the vector network analyzer detection system. Thus total input power and stability of the source and the detailed electronic characteristics of the detector become important boundary conditions in the design problem. Furthermore, since α_dielectric_ is also linear in the microwave frequency, eqn (25) suggests that the optimal length of the transmission cell – all other conditions being equal – will be inversely proportional to the microwave frequency.

Occurrence of other attenuation mechanisms brings the line diameter back into the cell design. Higher-order modes can be sustained in coaxial lines above a limiting microwave frequency: the cutoff frequency ν_c_. The first mode to appear is the TE_11_ mode whose cutoff is approximatively given by (e.g., [16])
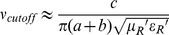
(26)


Since partial conversion of the TEM mode to higher-order modes is detected as loss, because they are largely reflected at the connection of the EPR cell to the detector system, the sum of cell radii a+b, and therefore the total diameter is physically limited especially for high-permittivity dielectrics such as water.

The TEM wave is solely transmitted in the dielectric (and dispersively in the paramagnet); penetration into the conductors only leads to resistive losses (e.g., ref. 12)
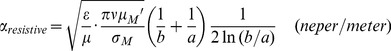
(27)in which ε and μ apply to the dielectric, µ_M_′ is the real permeability of the conducting metal, and σ_M_ is the metal’s conductivity in siemens/m. Note that resistive loss is proportional to the square root of the frequency, and is therefore usually outdone at increasing frequency by dielectric loss which is linear in the frequency. However, resistive loss is also inversely proportional to the line diameter, and will thus pose an excessive-loss limit (or even a limit of power breakdown by arcing) to miniaturization of the transmission cell.

Connection of two line segments with different characteristic impedance values leads to mismatch loss by reflection, which is usually defined (e.g., [13]) in terms of the voltage reflection coefficient, 0 ≤ |Γ| ≤ 1,
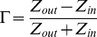
(28)as a positive return loss
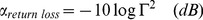
(29)Since we intend to replace the dielectric of a regular coax with a variety of (dilute) paramagnetic samples as solutions, powders, or frozen solutions, in a variety of dielectric containers, there will not be a simple, generic design solution to the minimization of return losses. A related problem is in the non-ideal geometry of the coax transmission cell itself where, e.g., variation of the excentricity of the inner conductor leads to a variation of characteristic impedance (eqn (13)), and thus to distributed reflections over the length of the cell.

Finally, the permeability of the paramagnet, µ_R_, in eqn (21) (and therefore α′ in the transmission eqn (23)) is of course a function of the quantum mechanical transition probability of the spin system. In conventional EPR spectroscopy the resonator geometry is chosen such that the microwave magnetic component B’ is perpendicular to the static field vector B, B’ ⊥ B, ensuring, e.g., maximal transition probability for S = 1/2 systems. Sometimes the parallel configuration B’ || B is employed predominantly to make otherwise forbidden transitions allowed in integer-spin systems [17]. The relevance of this choise of geometry for the present research lies particularly in the fact that neither one of these unique orientations is easily implemented with a coax cell in an electromagnet. Although we will explore a few simple examples of short-length cells with B’ ⊥ B, for reasons of sensitivity the majority of the developed transmission cells will have helical or helical-torroidal geometries, which implies a continuous variation of the angle between B’ and B over the propagation vector in the cell.

**Figure 2:**
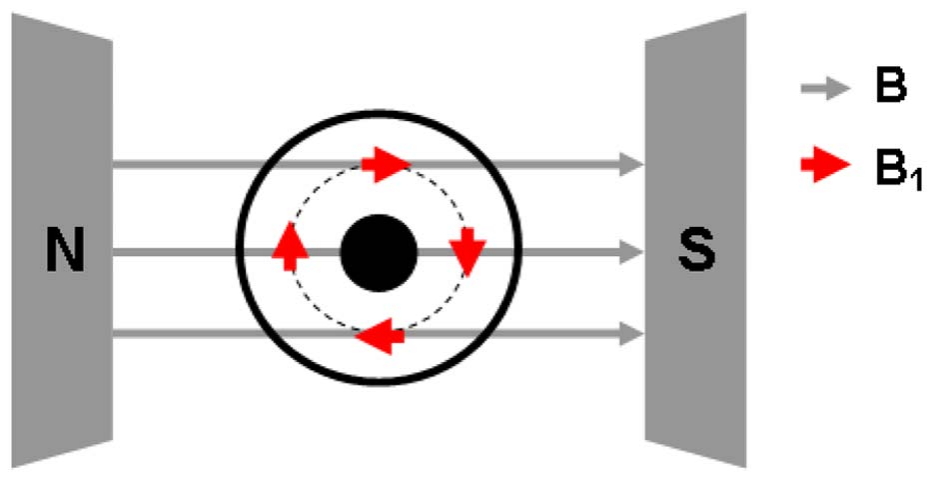
A coaxial structure with its TEM propagation axis perpendicular to the B-axis of an external magnetic field. The microwave magnetic-field component B_1_ in the sample compartment runs through all possible angles with respect to the external field vector B.

In S = 1/2 systems the transition probability, w, for B’ at an angle θ with respect to B is proportional to sin^2^θ [18], and, defining the transition probability for B’ ⊥ B as unity (w ≡ 1), a simple solution to the path-integrated EPR intensity is obtained as follo ws. For a straight coaxial segment whose propagation axis is colinear with the dipolar magnetic-field axis all insulator molecules experience B’ ⊥ B. When the cell axis is perpendicular to the B axis, as in Fig. 2, we must integrate the transition probabilitity w over a π/2 quadrant (or any multiple) of the circular path of B’ from B’ ⊥ B (unit intensity) to B’ || B (zero intensity) affording an average relative intensity of 1/2
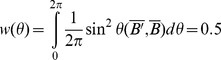
(30)


When a long coaxial is wound in a tight helix of small pitch and placed in the magnet with its helical axis perpendicular to B the axis of the propagation vector γ of infinitesimal slices thought the cell runs over an angle α from colinear to perpendicular and the integrated EPR intensity runs from unity to half unity, which can be formalized as:
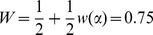
(31)in which
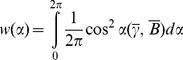
(32)


In other words for a fully filled helical cell the overall intensity is 0.75 of that of a linear cell of the same length coaxial with the external field. Furthermore, the intensity will not change when the helix is modified into a quasi-torroidal shape by bending the helical axis into a circle whose surface is perpendicualr to B. For partially filled cells with a filled sectorial fractional volume V eqns (30)–(32) generalize to
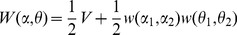
(33)which may be used to maximize certain B’ versus B orientations in the study of integer-spin systems.

If only a colinear subsection of the cell is filled (cf Fig. 1C), such as in a cell made up of a tube holding a liquid sample, then the relative power P_s_ (versus the total power P of eqn (22)) through this inner section is seen to be
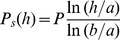
(34)which can be generalized for constructs with multiple segments as



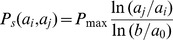
(35)In summary, the S = 1/2 EPR intensity in a TEM transmission experiment is defined by eqns (21), (22), (24), (33), and (35) as
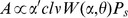
(36)in which α′ is the quantum-mechanical transition probablility of the S = 1/2 system, c is the spin concentration, l is the length of the cell (e.g. measured along its conductors), υ is the microwave frequency, W is the correction factor for filled sectors (W = 0.75 for completely filled cells), and P is the correction factor for filled sub-cylinders (P = 1 for samples contained only by the outer conductor).

### Transmission EPR Spectrometer

**Figure 3:**
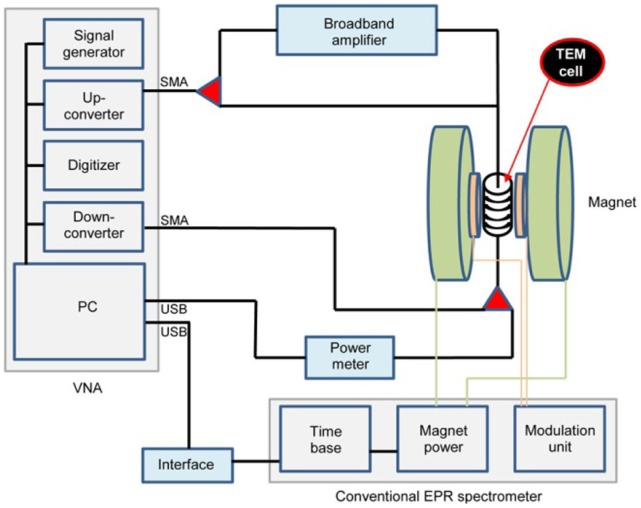
Outline of the transmission EPR spectrometer. An arbitrary microwave produced in the vector network analyzer (VNA) is send – either directly or via a broadband amplifier – to a transmission cell placed in the electromagnet of a conventional EPR spectrometer. The sample-attenuated output of the cell is either detected in a broadband power meter or as a carrier amplitude by the VNA. The time base of the magnet is used to trigger the onset of signal detection. The modulation coils of the magnet are employed in rapid-scan EPR.

An archetypical transmission EPR spectrometer consists of a microwave source, a coaxial transmission cell placed in the field of a magnet, and a microwave detector. In our prototype spectrometer (Fig. 3) the source is a National Instruments PXI-5641 16-bit 100 megasignals/s digital signal generator and a PXI-5610 superheterodyne upconverter affording 0.25–2700 MHz output of arbitrary shape and up to 12 dBm output power, followed by a Milmega series-2000 broadband (800–2700 MHz) RF multi-stage amplifier providing circa 50 dB power increase up to maximally 15 watt.

The transmission cells are (diluted) paramagnet-filled coax line segments whose dimensions in the TEM propagation direction are limited by the homogeneous-field volume of the dipole magnet. We used various electromagnets available from our conventional EPR spectrometers, namely (i) a Varian 9 inch magnet from our S-band spectrometer with a wide gap of 91 mm after removal of poles for short straight cells whose propagation direction can be lined up as || B, (ii) a Bruker BH 11-D magnet from one of our X-band spectrometers with a gap of 59 mm and a 15 cm diameter homogeneous-field circle to take helico-torroidally wound cells with a total propagation length up to circa 12 m, or (iii) a Varian 9 inch magnet with tapered pole caps from our Q-band spectrometer to accommodate helically wound cells of propagation length limited to circa 40 cm for rapid-scan EPR using the Q-band modulation coils that produce a rapidly time-varying homogeneous field in a cylindrical volume of 39 mm radius and circa 3 cm height.

The detector is a National Instruments PXI-5600 downconverter and the 100 megasignals/s PXI-5142 IF digitizer for 0–2700 MHz and +20 dBm maximal unattenuated input. Together with a controling PC the source and detector units are integrated in a NI PXI-1042 chassis with 132 MB/s PCI busses, thus forming a complete digital vector network analyzer (VNA). The downconverter carries an oven-stabilized 10 MHz onboard precision reference clock also serving the digitizer through a matched coaxial line and the signal generator units through the timing and triggering chassis busses.

The scanning electromagnets are regulated using the time base units of the standard spectrometers whose start pulses are fed into the controlling program running on the embedded PC via a National Instruments USB-6221 interface. In one setting the Bruker ER 001 time base is ran at its fastest scan rate of 10 s and a second spectrum is recorded when the field returns instantaneously also in 10 s. With an intrinsic start up delay time of circa 5 s this mode of operation affords averaging of 292 single scans in one hour with only slight field oscillations at the extremes of the field scan. The high frequency stability of the VNA allows massive data acquisition over several days (e.g., over 10,000 double scans in 3 days).

In contrast to regular EPR spectroscopy no modulation of the static field is employed, which opens the possibility to rapidly scan the field for increased data collection rates [19]–[22]. For lack of dedicated equipment we emulated a rapid field scan unit using the cylindrically shaped modulation coils of our Varian Q-band spectrometer operated at 35 Hz and 40 gauss nominal peak-to-peak modulation amplitude. The unit was operated independently (no triggering) and transmission EPR data collected at maximal rate by the VNA were subsequently –phase-synchronized in a dedicated computer program.

The source unit is typically programmed to produce a monochromatic sine wave from the 0–2.7 GHz range, which is amplitude modulated to a dept of 80% at a frequency of 25 kHz with an IQ-data generation rate of 625 kHz and a bandwidth of 500 kHz. After passing through the transmission cell the signal is double side band demodulated in the detector unit at an IQ-data detection rate of 1 M signals/s and the carrier wave amplitude is recorded in Volt units.

In an alternative setup we replace the detector system with a National Instruments USB-5680 power meter for 0–6 GHz or the USB-5681 power meter for 0–18 GHz. With this broadband detector the sensitivity (signal-to-noise ratio) drops by 2–3 orders of magnitude, but we now have a simple setup to monitor insertion losses (intrinsic and/or mismatch) of transmission cells. We also have a simple and economic, be it not very sensitive, way to extend transmission measurements to the higher frequencies provided by the microwave bridges of our standard spectrometers, namely 3.8–4.1 in S-band and 9.1–10.0 GHz in X-band. The Bruker S-band bridge has an coaxial SMA exit port, and the waveguide port of X-band bridges can be fitted with a waveguide-to-coax adapter.

### Cells for Transmission EPR

Conceptually the simplest possible cell is a piece of coax ending on both sides in lossless connectors and with the paramagnetic sample completely occupying the insulator space in between the inner and outer conductors. For EPR the cell should be placed in the dipolar magnet colinear with the field B in order for the microwave magnetic component B’ to be perpendicular to B as required for maximal transistion probability of regular |Δm_S_| = 1 EPR. This limits the length of the coaxial to less than the gap between the poles of the magnet.

**Figure 4:**
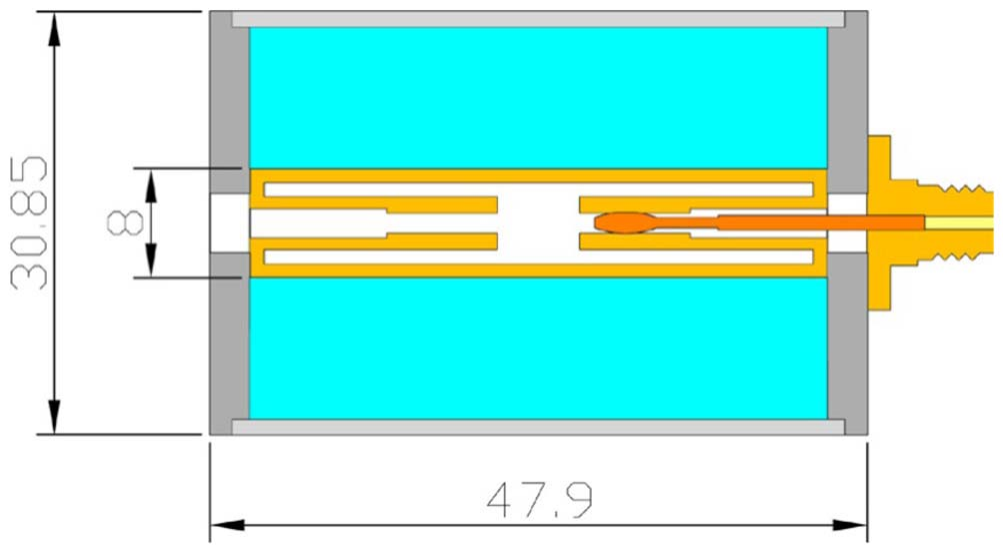
Drawing of the standard transmission cell. This cell was designed for ease of operation in particular for rapid sample change. Its short length, in combination with cables with right-angle male SMA connectors, allows orientation of the TEM axis parallel to the B-axis of a wide-gap magnet. The light-blue area is the sample compartment.

A practical design approaching this idealized simple case is shown in Figure 4 for use with an electromagnet with 92 mm air gap. A polyvinylchloride cylinder closed with side lids forms an easily demountable and cleanable holder for powder samples. Each lid is centrally pierced with a ‘screw on’ female SMA connector whose teflon insulation is trimmed away nearly to the base plate with 1 mm remaining to hold a plastic ring to isolate the base plate from the cell’s oversized (with respect to the connector) inner conductor. The extending inner lead of the connector is provided with a coaxial spring that fits to the cell’s inner conductor made of a hollow silver cylinder with brass end fittings. The outer conductor is made of household aluminum foil tightly wrapped around the cell including the side lids and making electrical contact with the base plate of the connector. The ratio of the diameters b = 29 mm and a = 8 mm of the conductors was chosen for Z_0_ ≈ 50 Ω with a dielectric with relative permittivity ε_R_ ≈ 2.3, to accommodate ionic salts (2< ε_R_ <5) with reduced effective ε_R_ values when employed as fine-grained powders in air. No attempt was made to compensate for the small mismach from the step in conductor dimensions at the connector-cell interface. The cell is connected to the source and detector via right-angle male SMA connectors and 50 Ω coaxial cables. A range of teflon inserts allows for tests on the replacement of part of the paramagnetic powder sample from specific geometric areas of the cell. Note that we chose the cell radial dimension with a view to ease of handling only. The cutoff frequency is predicted (cf eqn (26)) to be at the high-end limit of our frequency range for ε_R_′≈3.7.

The cell can of course also be operated in other that coaxial orientation with respect to the external dipolar magnetic field. What this means, in particular, for perpendicular orientation is illustrated in Fig. 2. The orientation of the microwave B_1_ field changes from perpendicular to the external dipolar field (B’ ⊥ B) to parallel (B’ || B) over a quarter B_1_ circle. For EPR of S = 1/2 systems and half-integer spin S = n/2 systems in the weak-field limit (i.e. effective S = 1/2 systems) this means that the transition probability runs continuously from its full value to zero [3], and when integrated over all orientations the total spectral amplitude will be 0.75 of that obtained with the cell in coaxial orientation with the external field (eqn (31)).

In this geometry the cell is also suitable for the detection of EPR from non-Kramers systems with their strongly mixed ground spin manifolds producing |Δm_S_| = 0 transitions when B’ || B [1]. The resulting spectra will not be directly comparable to those obtained with the well-known parallel-mode rectangular X-band resonator [17] because the coaxial cell sustains the complete spectrum of all B’ versus B orientations.

**Figure 5:**
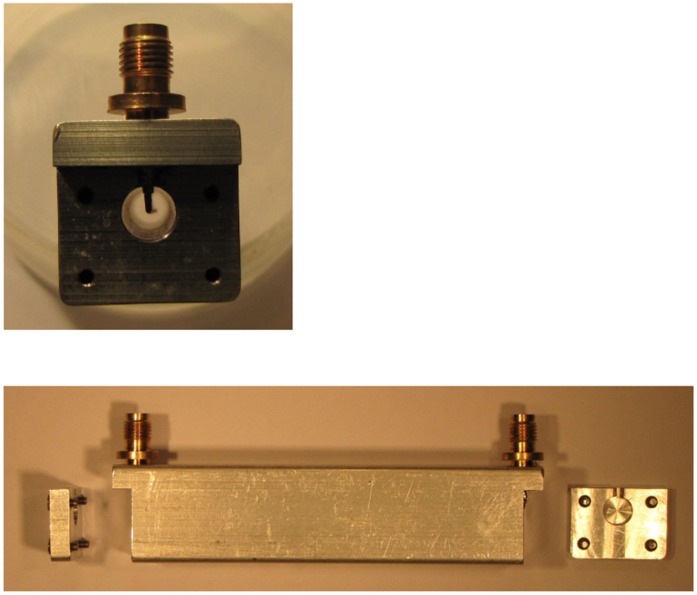
Picture of a monolitic aluminum cell. With its perpendicular SMA connectors this 82 mm inner length construct is the longest possible cell allowing TEM || B operation in our wide-gap magnet.

As a variant to the standard cell of Fig. 4 we also constructed a set of cells from aluminum as shown in Fig. 5. The solid aluminum functions as a holder and outer conductor to a machined cylindrical space in which a copper wire inner conductor runs, which is connected to two female SMA bulkhead connectors perpendicular to the cell body so that a maximal cell length of 82 mm is achieved. With the screwed-on closing plates of 4 mm thickness the whole cell just fits into the electromagnet’s 92 mm gap. To check (in)dependence of signal amplitude on coax diameter three cells were made with outer conductor inner diameters of 8, 6, and 4 mm and inner conductor diameters of 1, 0.7, and 0.7 mm, respectively.

To drastically increase the cell’s sensitivity we have its length as the only practical design parameter available. For the cell to fit in between the poles of the magnet we must bend it along the TEM propagation direction, and this means that we must use flexible materials. For cells to hold aqueous solutions we chose the following parts. For the inner conductor we used polymer film insulated copper wire of typically 1 mm diameter on which we apply a transversal pull by human force to somewhat reduce bending flexibility. For the container of the liquid we used flexible silicone rubber tubing of 55 Shore A hardness and typically 2 mm inner diameter and 4 mm outer diameter. After machining one end of the copper wire into a smoothly rounded tip, we could simply push the wire through the tubing for sections up to circa 2 m. For longer sections up to 12 m we pre-filled the tubing with household dishwasher detergent to reduce mechanical resistance. After placement of the wire the detergent was washed out with water and the cell was dried with filtered compressed air.

For the outer conductor we used 0.025 mm thick household aluminum foil cut into 4×30 cm strips, which we wrapped around the container at an angle of circa 70 degrees to create multiple overlaps.

**Figure 6:**
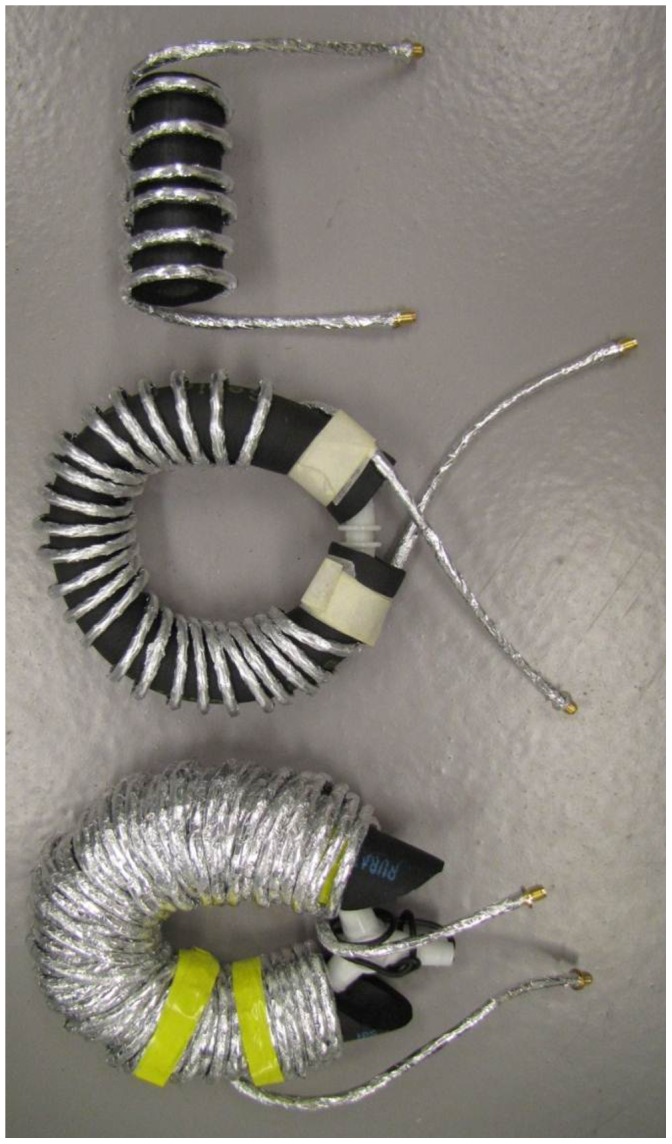
Long helical cells with continuously varying B_1_ versus B angle. The sample-compartment lengths are 75, 300, and 1215 cm, respectively. The coax is wound around a supporting piece of elastomer into a helix or a toroidal helix.

We then cautiously shaped the cell into a helix by subsequent wrapping around cylindrical templates of decreasing diameter from circa 12–3 cm diameter. To further confine the space taken up by the cell to the cylindrical box shape of the homogeneous field between the poles of the electromagnets we wrapped the helix around a piece of elastomer (copper pipe insulation of circa 35 mm o.d.) with a piece of insulated copper wire inside, and we carefully bended the helix into a quasi toroidal, or doughnut shaped, structure (Fig. 6).

To fill cells with dilute aqueous paramagnetic samples we used plastic syringes with pipette tips that we trimmed so that they would easily fit around the inner conductor wire and tightly fit into the silicone tubing. After removal of the syringe and filling the cell up to the rims with a Hamilton syringe we used 1 cm long tubing sections of i.d. = 1 mm and o.d. = 3 mm as end stoppers for the liquid container.

We removed the insulation from the copper-wire ends and trimmed them to be soldered to female SMA bulkhead connectors. The soldered connections and the stoppers were then put into a collar of transversally cut 2/4 mm silicone tubing, and the aluminum wrap was extended to reach the connector’s outer conductor. To replace a sample we had to unwrap and desolded the connectors, flush out the sample and wash the cell with water, and then re-fill and re-construct.

### Intensity Dependence on Cell Geometry

The S = 5/2 d^5^ system high-spin Mn(II) in solid MnSO_4_⋅4H_2_O has been used in early days of EPR history to record the very first S-band spectrum at 2.93 GHz using a cavity filled with 173 grams of the salt in powder form [23]. We re-visited this compound as a convenient carrier of strong paramagnetism. In the Theory section, above, equations were formulated describing the relative intensity of transmission EPR versus cell geometric properties and cell orientation in the external magnetic field. We employed the short standard cells described in the previous section in combination with the high EPR signal intensity from pure MnSO_4_ powder to check these predictions.

**Figure 7:**
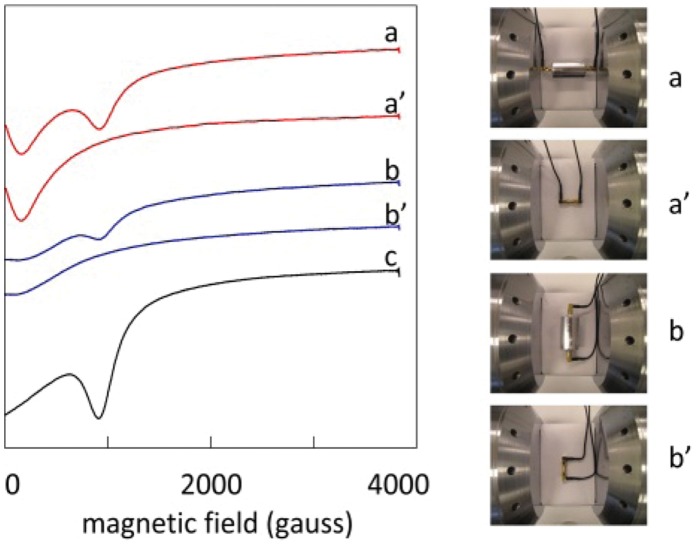
Transmission EPR of solid MnSO_4_⋅H_2_O in a 42 mm standard cell. Traces a and a’ are for TEM || B orientation, and traces b and b’ are for TEM ⊥ B. The primed traces a’ and b’ are baselines from short-circuited leads without transmission cell. Trace c is for the 82 mm aluminum cell in TEM || B orientation.

The 4.2 cm standard cell of Fig. 7 filled with ca 4 gram powder and placed with its TEM propagation axis parallel to the external magnetic field (therefore B’ ⊥ B) afforded the 2.7 GHz spectrum presented in Fig. 7a. The Mn(II) gives rise to a broad peak around g ≈ 2.00 at a resonance field of circa 965 gauss, i.e. hyperfine and zero-field structure is not resolved in the pure compound (cf ref. 23). A second peak of similar intensity and width is observed close to zero field, however this turns out to be a background signal because re-measurement after removal of the cell and short-circuiting the coaxial cables ending in right-angle male connectors with a female-female SMA connector, re-produces the low-field peak while the Mn(II) peak has disappeared (trace and picture a’ of Fig. 7). We then turned the cell over 90° to a position such that the propagation axis was perpendicular to the external field, and this creates a distribution of orientation between B’ and B from perpendicular to parallel as earlier depicted in Fig. 2. This resulted in the spectrum of trace b in Fig. 7 (and the baseline spectrum b’) in which both the Mn(II) peak and the low-field peak have reduced intensity. The last spectrum (trace c) was obtained with the 8.2 cm aluminum cell (4 mm diameter; 1.8 gram powder) in TEM || B orientation. The amplitude of the Mn(II) signal is increased because the cell is longer, and the amplitude of the baseline signal is decreased because the male connectors to the cell are straight and are perpendicular to the external field (Fig. 3–2).

**Figure 8:**
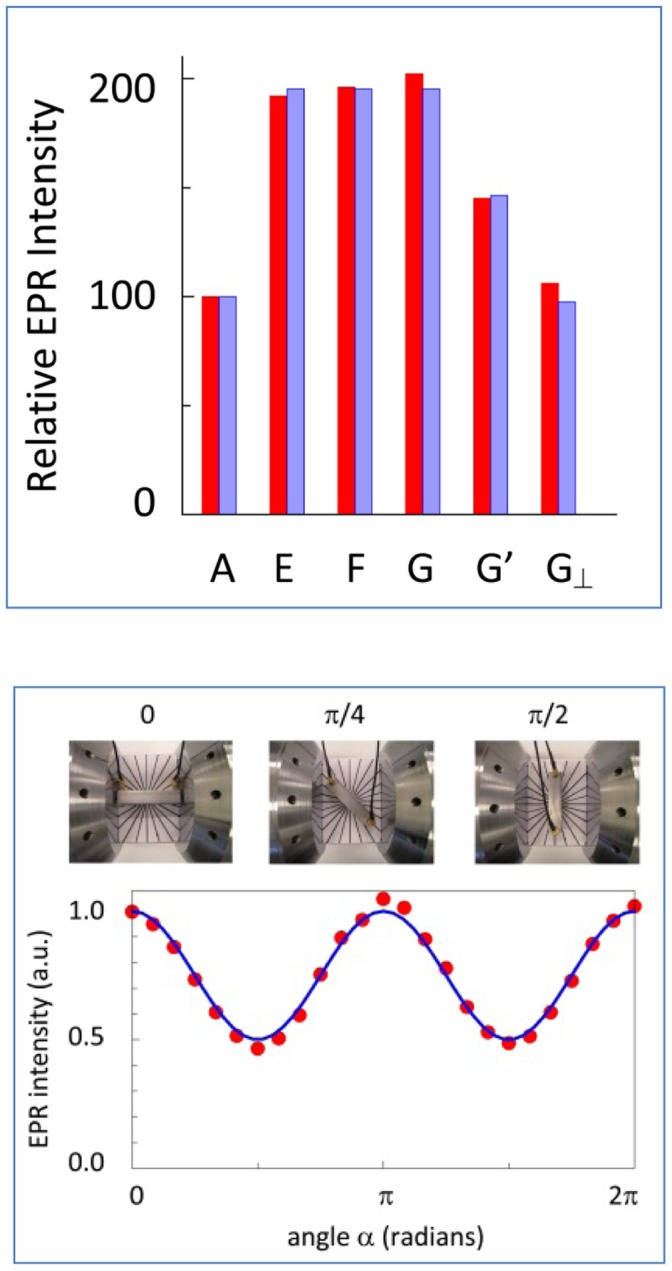
Signal intensity as a function of sample-compartment geometry. The 42 mm standard cell is partially filled with teflon spacers to displace paramagnetic sample. Bar A is the Mn(II) signal amplitude for a fully sample-filled cell in TEM || B orientation. Red bars are measured intensities and blue bars are theory-predicted. Subscript ⊥ indicates TEM ⊥ B orientation. In B a teflon spacer occupies half of the cell; in C the spacer is a cylinder around the inner conductor over the full cell length; in D two sector spacers occupy opposite positions in ‘butterfly’ orientation along the full length of the cell. The lower panel shows a full 360 degree rotation experiment for the ‘butterfly’ setup with intensity data fitted to eqn (30).

This qualitative monitoring of intensity was made quantitative in the following experiments. As seen in the upper panel of Fig. 8, when the standard cell was rotated over 90 degrees the signal intensity halved, and when the sample volume was halved by insertion of a teflon filler the intensity was also halved. A different filler over the full length of the cell but radially covering only the space from the inner conductor halfway towards the outer conductor afforded a relative intensity of 36% (experiment C) consistent with eqn (34).

A more complex sample-space displacement was realized with the ‘butterfly’ shaped filler covering two opposing 90° sectors, or quadrants, minus the space occupied by the inner conductor (experiment D), which also resulted in a volume reduction by half. With in-line orientation (TEM || B; B’ ⊥ B) the Mn(II) signal was simply halved compared to the fully filled cell. However, when the propagation axis was set perpendicular to B (resulting in the B’ versus B orientation distribution of Fig. 2) the EPR intensity depended on the orientation of the sample quadrants with respect to this distribution. With the sample sectors closest to the poles of the magnet the intensity was maximal (37.4% according to eqn (30) integrated twice over the interval −π/4 to π/4 ) while rotation over 90 degrees afforded minimal intensity (12.6%). A complete angular dependence of this effect over a full circle rotation is given in the lower panel of Fig. 8.

**Figure 9:**
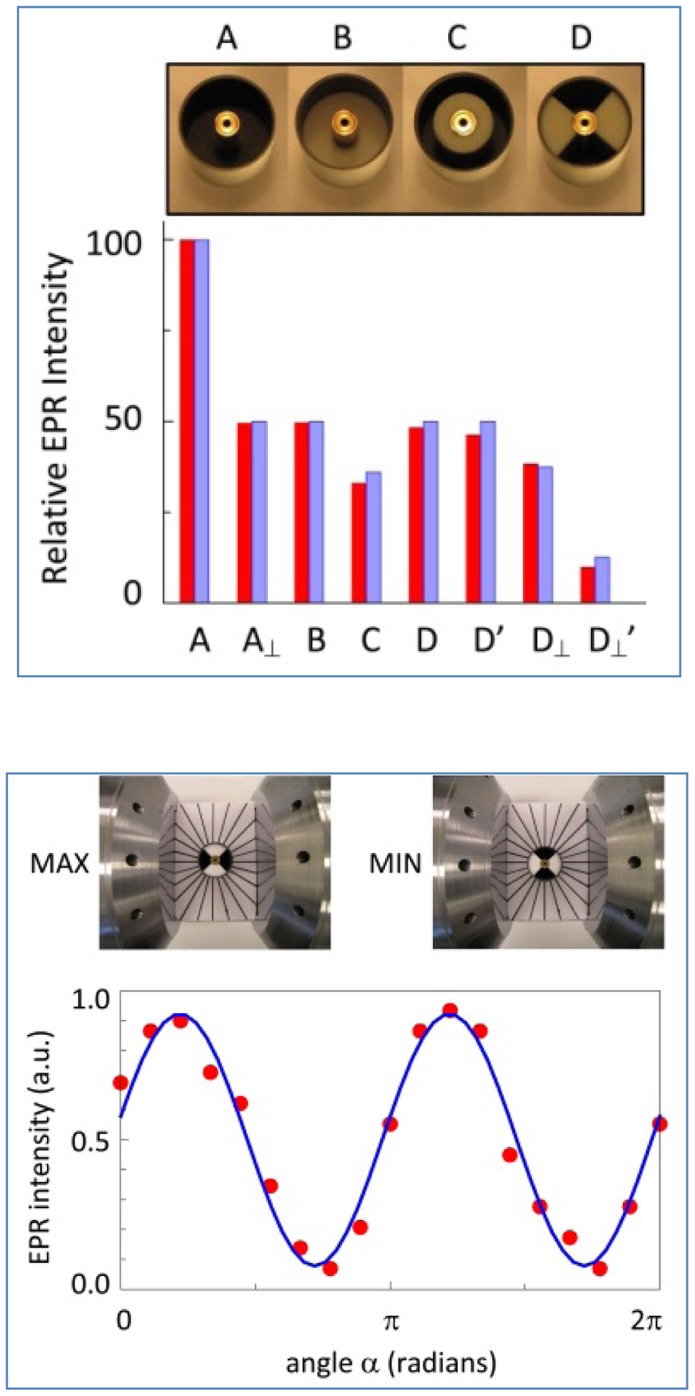
Signal intensity as afunction of TEM versus B orientation. Bar A is the Mn(II) reference signal from the 42 mm standard cell. Red bars are measured intensities and blue bars are theory-predicted. Bars E, F, and G are from 82 mm Al cells with inner diameter of the outer conductor 8, 6, and 4 mm, respectively. Bars G’ and G_⊥_ are for the 4 mm i.d. cell with TEM axis at 45 and 90 degrees versus B. The lower panel shows a full 360 degree rotation experiment for the 4 mm i.d. cell with intensity data fitted to eqn (30).

In the upper panel of Fig. 9 the intensity of the Mn(II) signal is seen to be linear in the length of the cell when the standard 42 mm cell is compared to three different 82 mm aluminum cells consistent with eqn (22). In contrast, the signal is independent of the diameter of the aluminum cell over the range 4–8 mm. Rotation of the aluminum cell over 45 and 90 degrees away from the in-line orientation afforded an amplitude reduced to 75 and 50%, respectively consistent with eqn (30). A complete angular dependence of this effect over a full cricle rotation is given in the lower panel of Fig. 9.

### Integer-spin Transmission EPR

The low-field background signal in Fig. 7 originates in the male SMA connectors. Removal of the female-female connector and short-circuiting the males with a small wire inner conductor and an aluminum foil wrap outer conductor led to a large impedance-mismatch loss, but the signal persisted. The signal was maximal when the right-angle connectors had their connecting part parallel to B. The same signal was obtained with straight male connectors, shortened with a female-female elongation connector, with their propagation axis parallel to B. When the cheap, brandless connecting cables were replaced with high-quality cables, namely male-to-right-angle-male assemblies with 36 inch LMR240 optimally for 1–4 GHz or with 24 inch HS086 optimally for 6–18 GHz (Fairview Microwave Inc, Allen, Tx), very similar spectra were obtained from right-angle and from straight connectors.

Electrical contact pins in SMA connectors are frequently made of high conductivity beryllium copper alloy containing significant amounts (of the order of 2%) of nickel; also, other parts of SMA connectors can be electroplated with nickel. Nickel atom has electronic configuration [Ar]3d^8^4s^2^, and nickel metal is ferromagnetic. Broad Ni(0) EPR signals have been observed, e.g., from nickel-plated brass [24], from nickel nanoparticles in La_2_NiO_4_ [25], from metallic nickel powder in KCl [25], and from annealed silica glass plates implanted with ^58^Ni^+^ [26].

**Figure 10:**
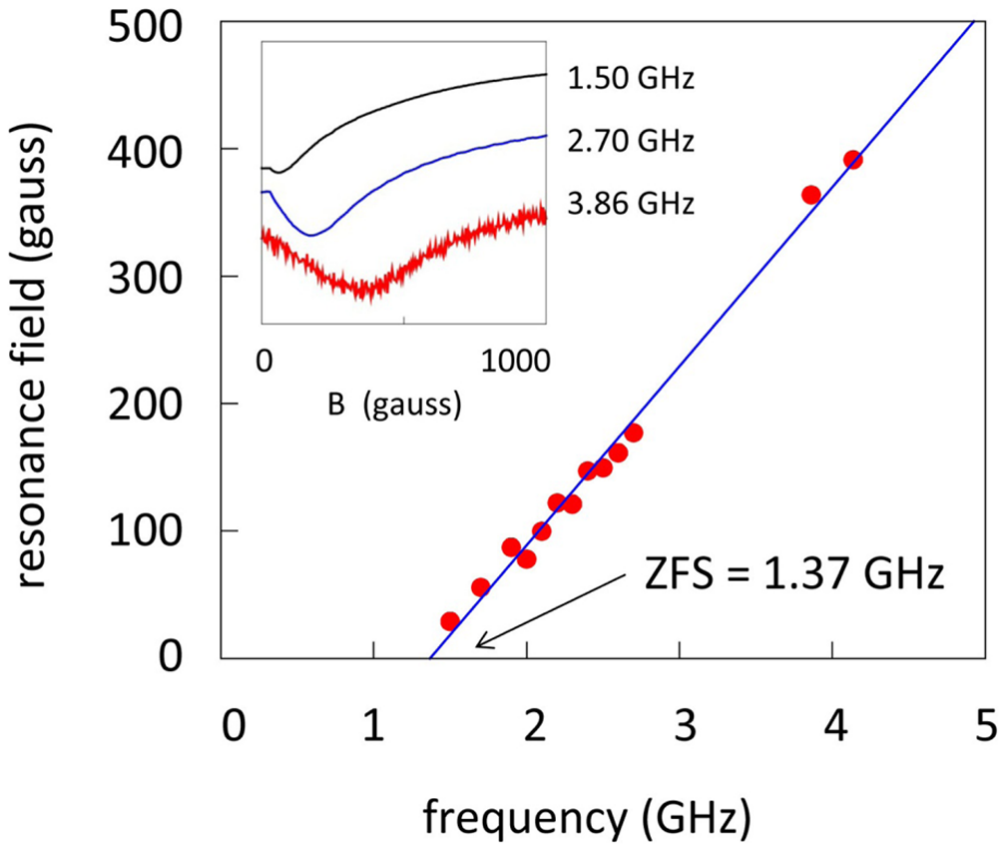
Frequency-dependent EPR signal assigned to S = 1 Ni(0) in male SMA connectors. The minimum of the broad signal (determined as the zero crossing of the derivative) was followed with frequency decreasing in 0.1 GHz steps until disappearance in zero field. Two points around 4 GHz were obtained with an S-band bridge as source and power-meter detection; two points at 9.1 and 10.0 GHz obtained with an X-band bridge (not shown) were also on the straight-line fit that extrapolates to a zero-field splitting of 1.37 GHz or 0.0457 cm^−1^.

We assign the baseline signals also to Ni(0) as the only reasonable candidate for magnetic material in SMA connectors. Ni(0) EPR has to our knowledge never been interpreted in terms of a spin Hamiltonian. From the multi-frequency experiment in Fig. 10 it is clear that the spectrum is from a transition that moves into zero-field at 1.37 GHz. With reference to the electronic ground state of Ni(0) a reasonable interpretation of this data would be an S = 1 triplet with axial zero-field splitting D = 1.37 GHz and an extensive distribution in spin-Hamiltonian parameters g, D, E. In a broader perspective the experiment of Fig. 10 suggests that transmission EPR is potentially a useful novel approach to the study of integer-spin systems.

### Doped-powder Spectra

The transmission EPR of solid powders in long helical cells should be not as challenging as aqueous solution experiments because dielectric losses will be strongly reduced due to their smaller ε_R_′-values of circa 2–5. As a test we diluted the MnSO_4_ H_2_O of the experiments in Figs (7–9) to 0.2% in the diamagnetic host ZnSO_4_⋅H_2_O and we filled a 220 cm long cell with 5 g of the doped material (i.e. 10 mg of MnSO_4_). The cell was wound in a helix of 14 cm heigth and 32 mm outer diameter with elongations on both ends of unfilled sections in order to keep the SMA connectors outside the magnetic field. No Z_0_-optimization was attempted.

**Figure 11:**
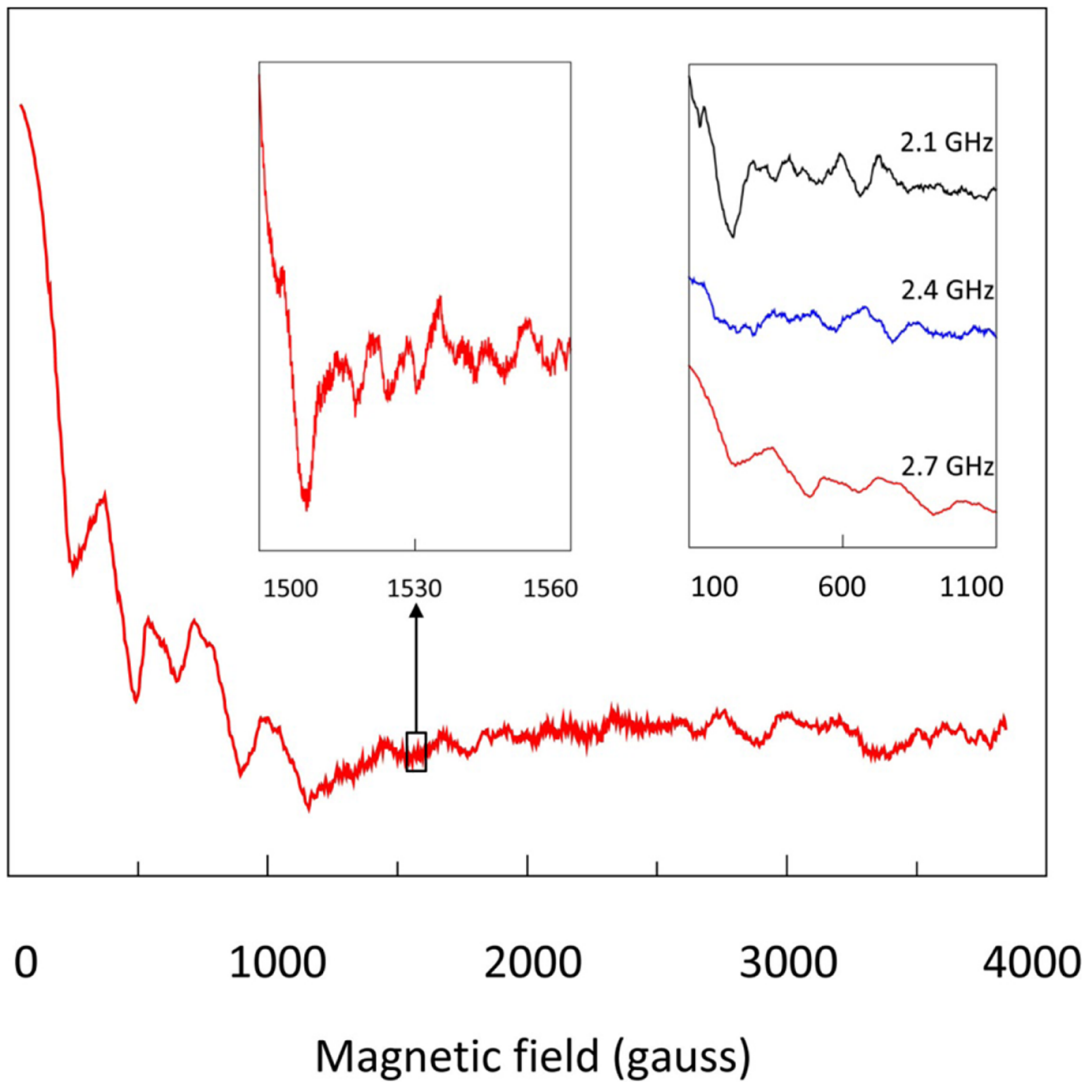
High-resolution transmission EPR spectra of 0.2 mol% Mn(II) in ZnSO_4_⋅H_2_O. The 220 cm cell held circa 5 g of powder of which circa 10 mg originated from MnSO_4_⋅H_2_O. Each trace is the differentiated average of circa 2500 forward scans of 100 s. The main trace was taken at 2.7 GHz; the left insert is a blow-up of a small section to illustrate detection of fine spectral details. The right insert shows very strong frequency dependence of the low-field part of the spectrum. Notethat these spectra are sums of spectra for all possible orientations of B’ versus B.

In a 4000-gauss scan at 2.7 GHz the single, broad line (Fig. 7) of pure MnSO_4_⋅H_2_O now resolves into a very complex (first derivative) spectrum (Fig. 11) with hundreds of lines and widely varying intensity. The spectrum is also strongly dependent on the frequency (inset to Fig. 11). The complexity is expected since Mn(II) in similar ionic hosts exhibits hyperfine and zero-field interaction strenghts of the order of 10% of the Zeeman interaction at X-band [27], [28], and so all three interactions will be of comparable magnitude at the frequencies employed here. Analysis will require development of appropriate software in which the energy matrix for the spin multiplet is diagonalized for all orientations of B’ versus B, but the results in Fig. 11 attest to the potential of transmission EPR for the collection of high-quality multi-frequency data of high-spin systems, be it – for the time being – at the expense of long data-collection times: each spectrum in Fig. 11 is the averages of circa 2500 scans of 100 s, i.e. some three days of measuring time per spectrum.

### Rapid-scan Transmission EPR

Transmission EPR spectroscopy is not yet competitive with regular cw-EPR spectroscopy in terms of sensitivity. One of the possible options for improvement would be an increased data acquisition rate. Rapid-scan EPR has been proposed as a modification of regular EPR initially at 250 MHz [19], [20] and subsequently at X-band [21], [22]. The concept involves replacement of magnetic-field modulation at 100 kHz and phase-sensitive detection by rapid field scanning over a few gauss at a rate up to 50 kHz and direct unmodulated detection of the EPR signal. Since in our transmission EPR setup no magnetic-field modulation is employed, rapid scanning of the field is readily implementable, and the amplitude-modulated detection scheme is not affected.

**Figure 12:**
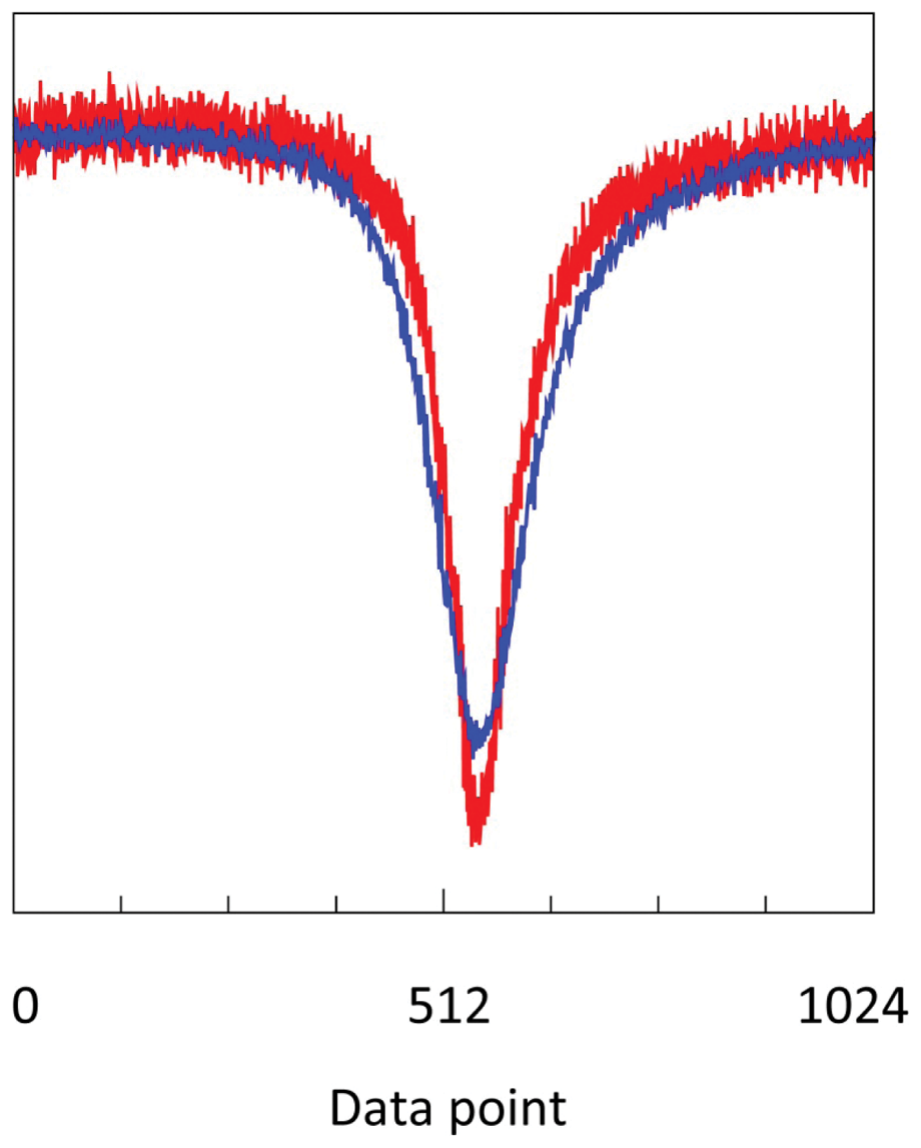
Rapid-scan transmission EPR to improve signal-to-noise ratio. A thick outer wall 40 cm cell was filled with solid DPPH, and a 2.7 GHz single-scan spectrum was taken in 30 s over a 959±20 gauss field range using a low-intensity microwave of −60 dBm incident power from the VNA to the amplifier to produce a spectrum with visible noise (red trace). Then a second spectrum was taken with the field at 959±0 gauss but with the field modulation unit set to 35 Hz and with a nominal peak-to-peak modulation amplitude of 40 gauss. The resulting data were mapped with a dedicated LabVIEW program to a sinusoidallly varying field and then interpolated to a 1024 point spectrum (blue trace). The increased width of the blue trace shows that the actual modulation amplitude felt short of 40 gauss, which illustrated that this experiment can be used as a convenient way to calibrate modulation coils. The rapid-scan EPR blue trace has reduced signal-to-noise (see text for details).

For proof of principle we constructed a 40 cm thin cell filled with 2,2-diphenyl-1-picrylhydrazyl (DPPH) stable radical, tightly wound in a small helix to fit inside the cylinder carrying the coils of a Varian Q-band modulation unit. Single scans of 30 s over 40 gauss afforded very strong signals, and the source power before injection in the broadband amplifier had to be reduced to −60 dBm (i.e. 1 nanowatt) to produce a spectrum with significant noise (red trace in Fig. 12). Subsequently, the slow field scan was switched off, a ±20 gauss modulation was applied at 35 Hz using the Varian E-line low-frequency modulation unit, and the amplitude-modulated carrier wave value was collected for 30 s. Then the data were mapped with adjustable phase by visual inspection onto a 40 gauss field sinusoidal sweep to produce the blue trace in Fig. 12. The increased width of the DPPH signal is due to the fact that the actual modulation field fell short of the set value of ±20 gauss, and was measured with an AC gauss meter to be ±16.4 gauss. The 35 Hz scan produces 1050 spectra in 30 s, which would imply a signal-to-noise increase of √1050 ≈ 32. However, the maximal data collection rate of the present setup is 60,000 points per 30 s while for the slow scan 3000 points were collected, and so the effective signal-to-noise increase is only √20 ≈ 4.5. In summary, rapid field scanning is a relatively simple addition to improve signal-to-noise ratios towards regular cw-EPR values, but this will require increased data collection rates through improvements in the VNA hardware and software.

### Aqueous Solution Transmission EPR

The most challenging transmission-EPR experiment by far is the detection of signals from paramagnets dissolved in aqueous solutions. Water has a high complex electric permittivity at RF frequencies and so the EPR effect is measured against a massive background of non-resonant absorption of microwaves. In regular EPR this problem is tackled by choosing a sample geometry in which overlap with the electric RF component is minimized, but in the fundamental TEM mode of coaxial structures electric and magnetic RF components are inseparable, which makes high power loss per unit length of transmission line unavoidable. Furthermore, with the high water value of ε_R_′ ≈ 80 and the boundary condition Z_0_ ≈ 50 Ω, filling the sampe area of a coaxial completely with water leads to impractical radial dimensions according to eqn (12). For example, taking µ_R_′ ≈ 1, an outer cell diameter of 4 mm would require an inner conductor with a mechanically and electrically unrealistic diameter of 0.0023 mm.

**Figure 13:**
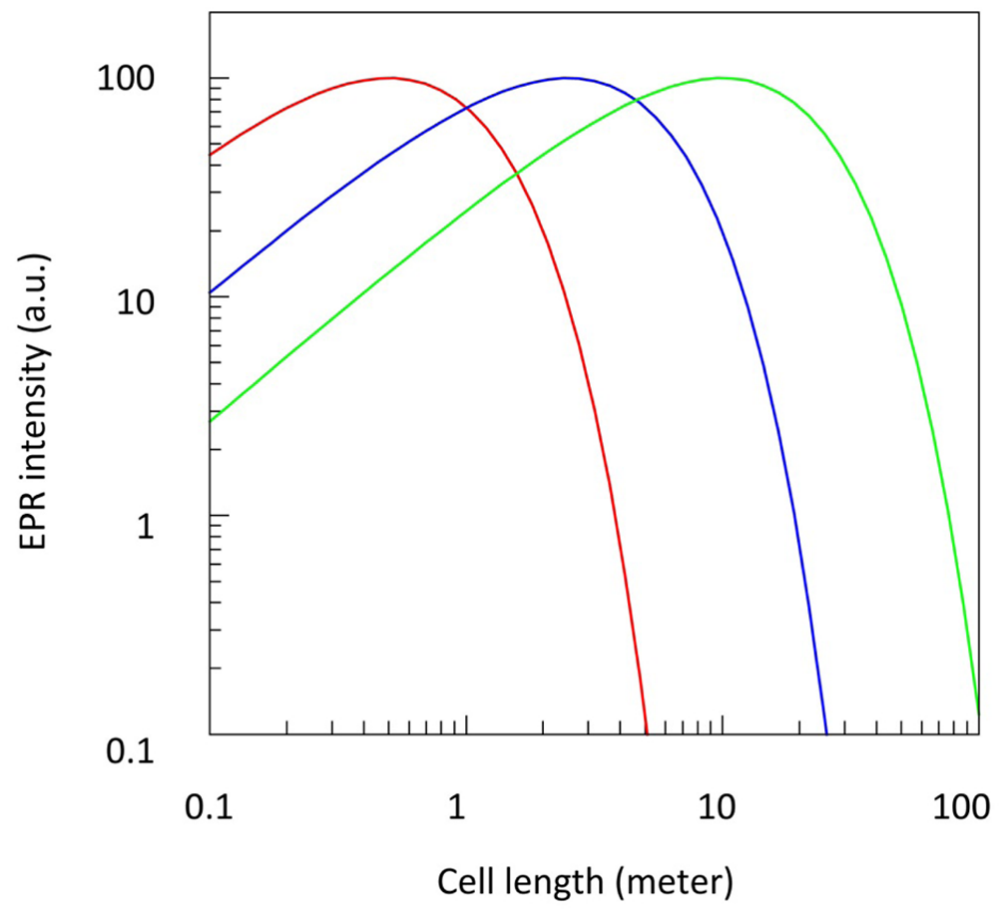
Transmission EPR signal from samples in lossy media as a function of cell length. These theoretical curves are based on eqn (37). Loss in dB/m is 40 (red), 8 (blue), or 2 (green). Maximal amplitudes are normalized to 100%.

We addressed this problem by filling up a major part of the total sample space in between the conductors with diamagnetic tubing to enclose an aqueous solution (Fig. 1C), which afforded an overall Z_0_ of circa 50 Ω even with an inner conductor of 1 mm diameter. The response of the coaxial transmission cell as a function of its length is now a trade-off between increasing power absorption by EPR versus increasing power loss by dielectric dissipation. Thus, for a given cell construction (material and dimensions) operated at a fixed microwave frequency and a fixed insertion power level the cell exhibits an optimum in the signal-to-noise ratio as function of its length as illustrated in Fig. 13, in which it is assumed that EPR absorption is linear in the cell length and the dielectric power loss in dB per meter has an exponential detrimental effect on the EPR signal
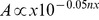
(37)in which x is the length in meters and n is the attenuation in dB per meter. Note, however, the schematic nature of this illustration in which curve amplitudes have been adjusted to the same arbitrary value because their actual relation for three different cells would depend on many more paramaters than the few in eqn (37). Furthermore, actual signal-to-noise levels would be difficult to predict since noise from the signal generator is proportional to the insertion power (resulting in a constant EPR S/N ratio over a certain power range) and noise in the detector becomes dominant only below a treshold power.

**Figure 14:**
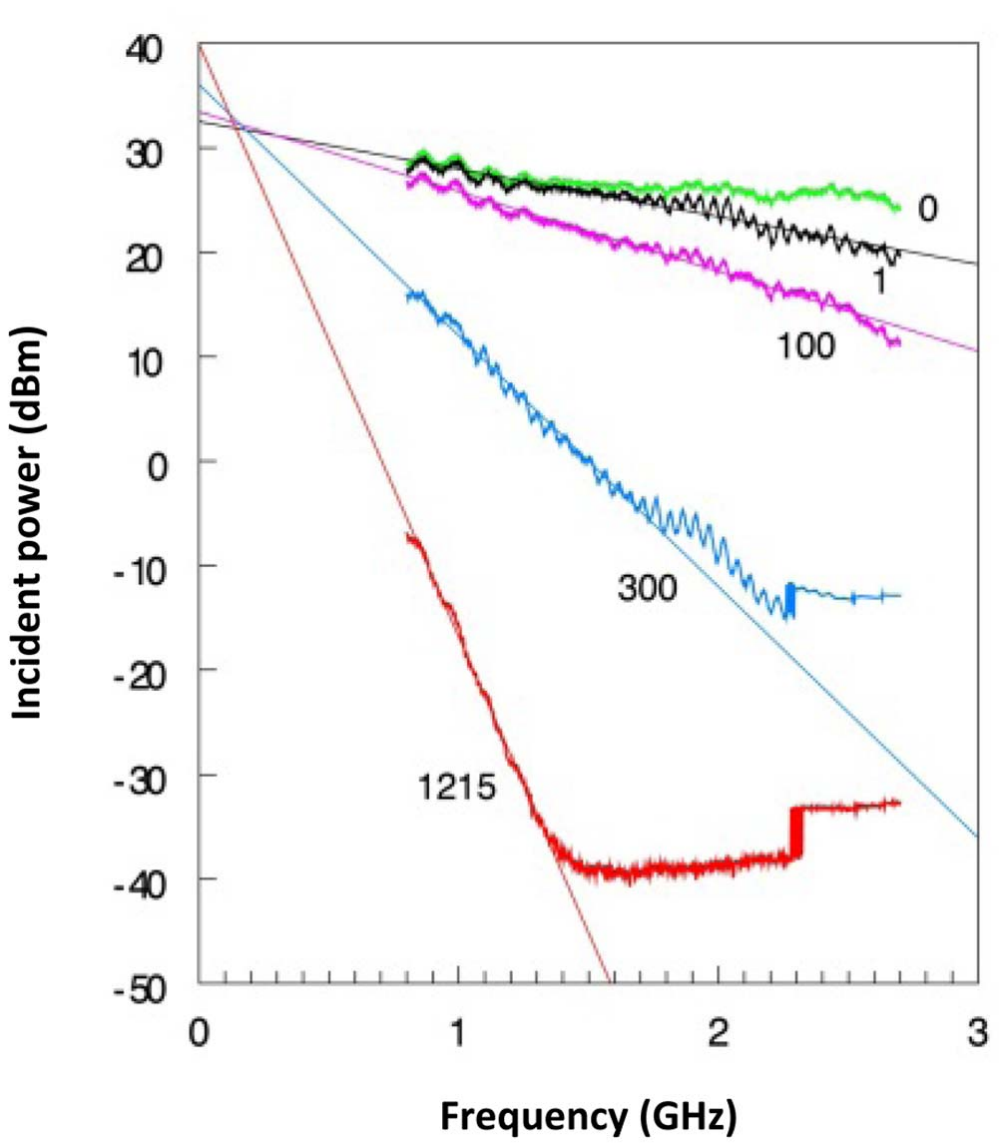
Power loss versus frequency in water-filled cells of different length. All cells were made of silicone tubing (4 mm o.d., 2 mm i.d.) with 1 mm diameter copper wire inner conductor (as in Fig. 6). Power from the VNA incident to the broadband amplifier was set at −30 dBm or, for the two longest cells, at −20 dBm. The three upper traces were shifted vertically over +10 dB to normalize all traces to −20 dBm incident power. The output power of the cells were measured with the broadband power meter. The green trace (0 cm) is for no cell and reflects losses in the brandless elongation cables of 1.5 m each. The black trace (1 cm) additionally shows losses due to impedance mismatch at the cell’s ends. Each trace was collected in circa 15 min as 1024 9-times averaged data points in the 0.8–2.7 GHz range.

Furthermore, eqn (25) predicts the loss to be proportional to the frequency. We can map this behaviour experimentally with cells of different length, filled with water, in which we insert RF at a fixed power and then measure the output power with a calibrated broadband power meter as a function of microwave frequency (Fig. 14). We found the loss in dB to be linear in the frequency with contributions from connecting cables (0 meter cell length), from impedance mismatch (0.01 meter), and from the cells themselves (1 to 12 meter).

**Figure 15:**
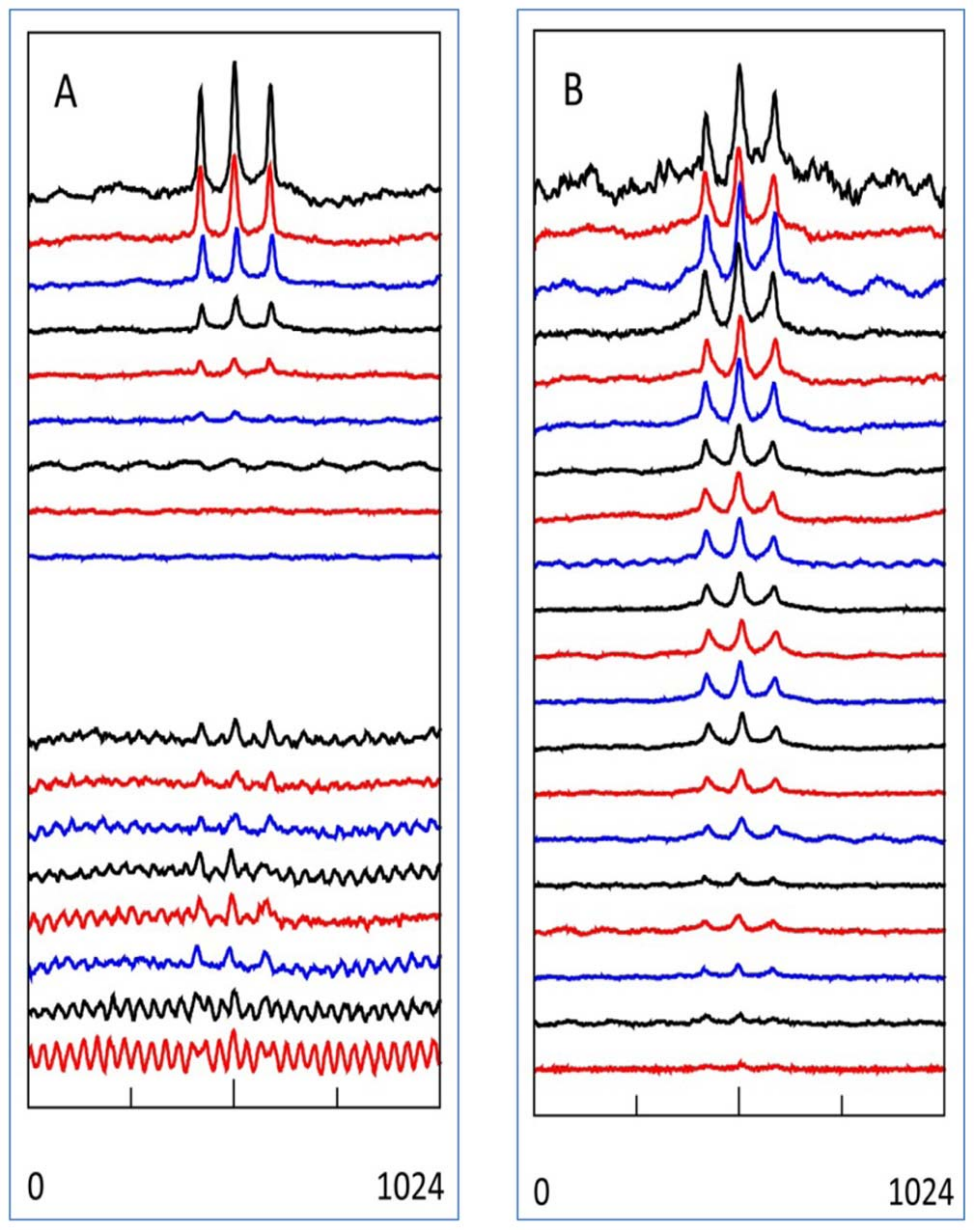
Dependence on frequency and on cell length of aqueous tempo tranmission EPR. Cells (cf Fig. 6) of 1215 cm (A), 300 cm (B) and 75 cm (C) were filled with 1 mM HO-tempo in 100 mM KCl. Spectra were collected at 0.1 GHz intervals from 0.8 GHz (upper trace) till 2.7 GHz (lower trace) as 500 averages of 10+10 s forward-reverse scans (i.e. circa 200 minutes per spectrum) over 200 gauss around the resonance field for g = 2 (i.e. 284±100 gauss for 0.8 GHz to 959±100 gauss for 2.7 GHz). In terms of maximal signal-to-noise the 12 m cell is seen to be optimal for frequencies approximately ≤ 1.1 GHz and the 3 m cell is optimal for circa 1.1–2.1 GHz. The 75 cm cell does not afford sufficient signal under the used conditions.

To explore how this complex trade-off between paramagnetic and dielectric absorption works out in practical detection limits of transmission EPR we measured the spectrum from a dilute aqueous solution of the nitroxide spin label HO-tempo (4-hydroxy-2,2,6,6-tetramethylpiperidine 1-oxyl) as a function of cell length and of microwave frequency. The S = 1/2 spectrum of Tempo and its derivatives in the rapid-tumbling limit at ambient temperature consists of an isotropic line split into a triplet by ^14^N (I = 1) hyperfine interaction and with a linewidth determined by multiple unresolved proton hyperfine interactions [3], i.e. a frequency-independent spectral shape. We constructed a long cell of 4 mm diameter and 12 m length, whose volume, when wound up in a toroidal helix, came close to the maximum that could be accomodated in the homogeneous-field space of the electromagnet with 59 mm gap and 17 cm pole diameter. The silicon-rubber tubing of the cell (1 mm wall thickness; and holding a 1 mm diameter inner conductor) was filled with 27 ml of an aqueous solution of 1 mM HO-Tempo and 100 mM KCl. Equivalent cells were constructed of length 3 and 0.75 m. Each cell was subjected to a constant RF power level such that incident power from the broadband amplifier was ≥ 20 dB below amplifier saturation and power reflected to the amplifier was negligible. Multi-frequency data with 0.1 GHz intervals were collected as 500 averages of 10+10 seconds scans (Fig. 15).

A cell length of 12 m turns out to be close to optimal for frequencies near the low-end limit of 800 MHz of the broadband RF amplifier. With increasing frequency the signal-to-noise ratio rapidly deteriorates due to dielectric loss of power (cf Fig. 15 A), and above 1.2 GHz it drops below unity. With a cell length of 3 m the spectrum is readily detected over the whole available frequency range of 0.8–2.7 GHz with a broad plateau of approximately constant S/N between circa 1.1 and 2.1 GHz. With another four-fold reduction in cell length to 75 cm this plateau appears to shift to higher frequencies, but the spectra suffer from low EPR signal intensity. As a general conclusion a cell length of the order of a meter appears to be optimal for application over a broad range of microwave frequencies, where the data in Fig. 15 predict – by extrapolation – applicability in terms of practical power levels up to X-band.

**Figure 16:**
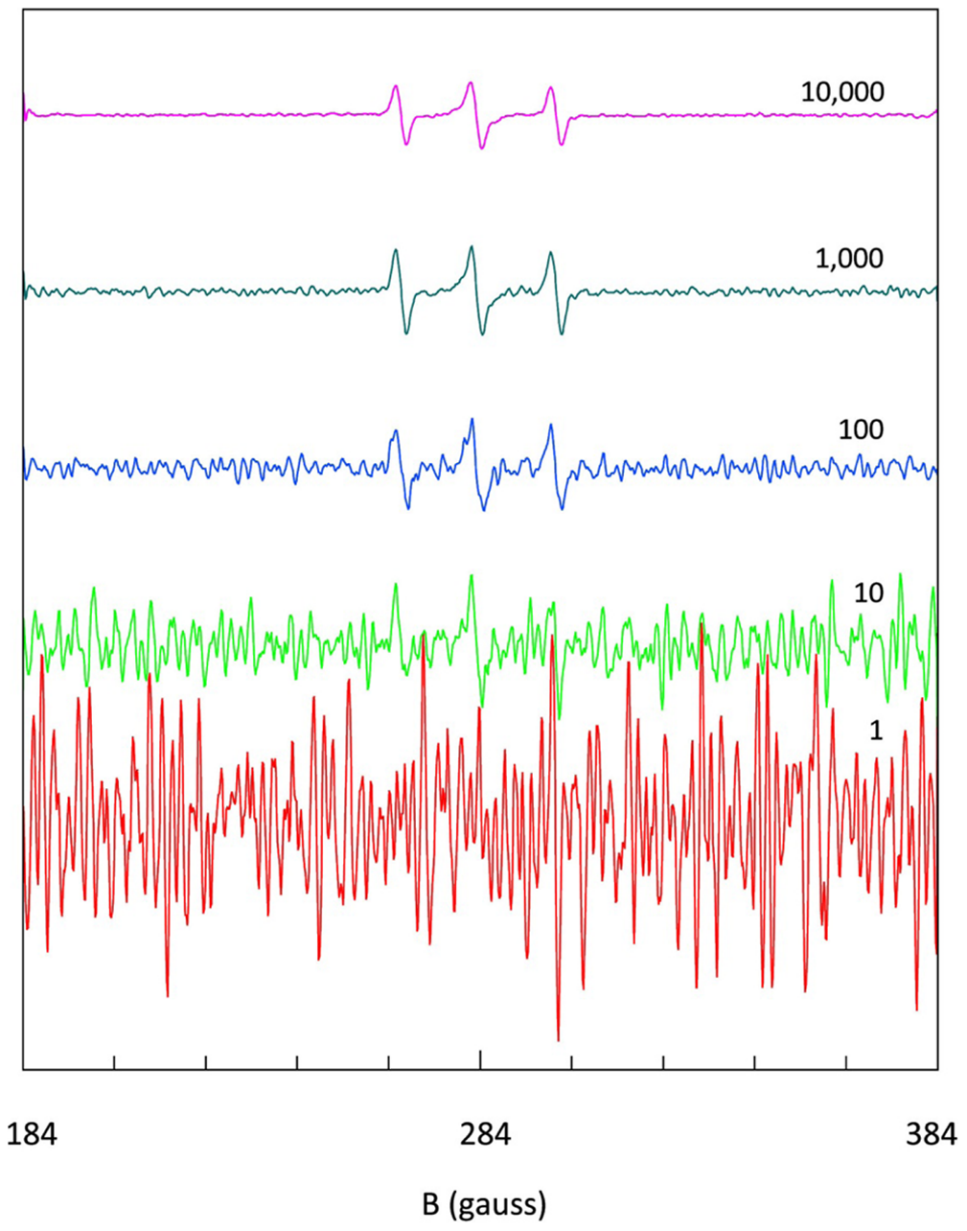
Estimation of tempo detection limit at 800 MHz. The 12 m cell was filled with 300 µM HO-tempo in 100 mM KC,l and increasing numbers of averages were collected of 10+10 s forward-return scans. From the differentiated spectrum of 10,000 averages (67 hours data colletion) a detection limit of circa 5 µM was determined.

To set a sensitivity standard for comparison with regular EPR and as a reference for further development of transmission EPR we signal-averaged the spectrum of 0.3 mM HO-tempo at 800 MHz in the 12 m cell up to 10,000 times. The spectra were collected as 20,000 point arrays, smoothed through a Savitzky-Golay filter (3-rd order polynomial) such that no resolution was lost after final convertion to 1024 point arrays, which were discretely differentiated (second order central method) to obtain the first-derivative EPR spectra in Fig. 16.

Signal-to-noise ratios in conventional EPR are generally determined by conversion of the maximum noise amplitude to RMS noise by division by a factor of 2.5 [29]. With this common definition we find for the present setup a transmission EPR detection limit (i.e. S/N = 1) of circa 5 µM HO-Tempo in aqueous solution at 0.8 GHz. From the data in Fig. 16 it can be seen that a similar detection limit is obtainable for frequencies up to at least 2 GHz with a 3 m cell.

## Experiment

Chemicals: manganese(II)sulfate monohydrate 99% and zinc sulfate heptahydrate ACS 99.0–103.0% were from Alfa Aesar, Karlsruhe, Germany. For the 0.2% Mn-doped zinc sulfate preparation 59 mg of the Mn(II) salt and 49.9 g of the Zn(II) salt were dissolved in water (Milli-Q; resistivity >18 MΩ⋅cm) to a total metal concentration of 1 M, and the solution was heated in a crystallizing dish to 130°C for 48 h. The residual solid was crushed to flakes with a spatula and once more heated to 130°C for 48 h, and then ground in a mortar to a fine powder with a yield of circa 31 g (i.e. the monohydrate). KCl pro analysi ≥ 99.5% was from Merck Chemicals via VWR International, Amsterdam, The Netherlands. HO-Tempo, or 4-hydroxy-2,2,6,6-tetramethylpiperidinooxy 98% free radical was from Acros Organics via Fisher Scientific, Landsmeer, The Netherlands. DPPH, or 2,2-diphenyl-1-picrylhydrazyl was from Aldrich via Sigma-Aldrich Chemie B.V., Zwijndrecht, The Netherlands.

Equipment has been described in detail, above, in the sections on Transmission EPR spectrometer and on Cells for transmission EPR.

### Conclusion

For nearly seven decades regular cw-EPR spectroscopy has been carried oud with single-frequency resonators usually combined with magnetic-field modulation. Comparison with broadband transmission EPR could incite a paradigm change both fundamentally (number of frequencies is unlimited) and practically (change of frequency is easy and cheap). In principle all molecular EPR spectra are frequency-dependent, and their rigorous analysis could benefit very significantly from the possibility of 2D (frequency-field) data collection with a single machine. The present work is an attempt to open up research into this field.

Compared with the few documented previous attempts to employ coaxial EPR cells the present setup appears to be a major improvement. Inspection of our 0.8 GHz three-line spectrum of 0.3 mM HO-Tempo in water (Fig. 16) suggests several orders-of-magnitude increase in signal-to-noise ratio compared to the six-line coaxial reflection spectrum of 700 mM manganese(II) in pure MnCl_2_⋅4H_2_O taken at 9.5 GHz [7]. Also, our 2.7 GHz spectrum of 0.2 mol% Mn(II) in ZnSO_4_⋅H_2_O (Fig. 12) has similar signal-to-noise but better resolution than the field-modulated zero-field spectrum of 1.5 mol% Mn(II) in Mg(NH_4_)_2_(SO_4_)_2_⋅6H_2_O taken as a frequency scan from 1 to 8 GHz [5].

Cylindrical resonators can be made frequency-tunable when constructed with moving parts, and this principle has been often applied in the past for tuning over a small bandwidth for example with P-band (circa 15 GHz) and Q-band (circa 35 GHz) cavities. A high-frequency broadband version (40–60 GHz) has been described for flat solid samples [30]. A broadband form has been recently described for tuning over a 14–40 GHz frequency range extendable down to 4 GHz by insertion of sets of dielectric plates [31]. The setup has been tested with pure Mn_20_ and V_6_ molecular magnets but sensitivity has not yet been documented for doped solids or for dilute liquid samples. It would be interesting to competitively compare this approach with the one proposed by us in terms of sensitivity and practical handling while the two methodologies develop.

In the present work signal intensity was found to be independent of radial cell dimension (at constant ratio of inner and outer conductor radii), but no attempt was made yet to minimize sample volume by decreasing cell radius. Miniaturization, possibly with lithographic technology, should not only increase absolute sensitivity, but it will also shift the cutoff frequency for loss via higher-order modes towards higher values. Significant future improvements in concentration sensitivity can be reasonably expected to be attainable with increased data-collection rates based on a combination of improvements in rapid-scan hardware, VNA hardware, CPU hardware, and in coding of software and programmable hardware. These options and also variable-temperature cryogenic applications are under our active investigation.

In summary, as a method under development transmission EPR in the present study has already shown value in certain nice areas in particular for half-integer and integer-spin systems with relatively small zero-field splittings. Further development towards a generally applicable spectroscopy should in our view be focused on improvement of sensitivity through rapid detection schemes, reduction of sample size through cell miniaturization, and extension towards higher microwave frequencies where properly constructed coaxial structures are in principle applicable up to circa 50 GHz.
